# Feasibility of quantitative sensory testing in juvenile idiopathic arthritis

**DOI:** 10.1186/s12969-022-00715-5

**Published:** 2022-08-09

**Authors:** Maarten O. Mensink, Niels Eijkelkamp, Dieuwke S. Veldhuijzen, Nico M. Wulffraat

**Affiliations:** 1 Pediatric Rheumatology, University Medical Center Utrecht, Utrecht University, Utrecht, The Netherlands; 2 Department Anesthesiology and Pain, Princess Máxima Centre for Pediatric Oncology, PO box 113, 3720 AC Bilthoven, The Netherlands; 3 Center for Translational Immunology, University Medical Center Utrecht, Utrecht University, Utrecht, The Netherlands; 4 Faculty of Social and Behavioural Sciences, Health, Medical and Neuropsychology Unit & Leiden Institute for Brain and Cognition, University Leiden, Leiden, The Netherlands

**Keywords:** ‘Quantitative Sensory Testing’, ‘Juvenile Idiopathic arthritis’, Pain, ‘Chronic pain’, Knee

## Abstract

**Objective:**

Juvenile Idiopathic Arthritis (JIA) is a childhood-rheumatic disease with pain as a major early complaint, and in 10–17% pain remains a major symptom. Very few data exist on sensory threshold changes at the knee in JIA, a location in which inflammation often manifests. We determined whether JIA is associated with sensory threshold changes at the knee by using Quantitative Sensory Testing (QST) and established reference values at the knee of children.

**Methods:**

Sixteen patients with JIA aged 9–18 years with one affected knee and a patient-reported pain by Visual Analog Scale (VAS) > 10 on a 0–100 scale, and 16 healthy controls completed the study and were included for the analysis. QST was assessed in compliance with the German Research Network on Neuropathic Pain (DFNS) standard. Disease severity was determined using Juvenile Disease Activity Score (JADAS. Perceived pain was assessed with a visual analogue scale(0–100). Feasibility of QST was tested in patients aged 6–9.

**Results:**

Under the age of 9, QST testing showed not to be feasible in 3 out of 5 JIA patients. Patients with JIA aged 9 and older reported an average VAS pain score of 54.3. QST identified a significant reduction in pressure pain threshold (PPT) and increase in cold detection threshold (CDT) compared to healthy controls. PPT is reduced in both the affected and the unaffected knee, CDT is reduced in the unaffected knee, not the affected knee.

**Conclusion:**

In a Dutch cohort of Patients with JIA, QST is only feasible from 9 years and up. Also, sensory threshold changes at the knee are restricted to pressure pain and cold detection thresholds in Patients with JIA.

**Perspective:**

This article shows that in a Dutch population, the extensive QST protocol is only feasible in the age group from 9 years and older, and a reduced set of QST tests containing at least pressure pain thresholds and cold detection thresholds could prove to be better suited to the pediatric setting with arthritis.

**Supplementary Information:**

The online version contains supplementary material available at 10.1186/s12969-022-00715-5.

## Introduction

Juvenile idiopathic arthritis (JIA) is the most common childhood rheumatic disease [1]. JIA is characterized by chronic joint inflammation, and can result in long-term disability that persists into adulthood in more than one-third of the patients [2, 3]. Pain in Patients with JIA is common and often persists for months to years [3–5]. Chronic pain is one of the major complaints of Patients with JIA and severely affects quality of life [1, 6–8]. Moreover, Patients with JIA have reduced pain thresholds compared to healthy subjects that persist after the inflammation in the joints had subsided [9, 10]. Persistent pain is difficult to treat. Literature on socioeconomic consequences in childhood is lacking, but in an adult setting, persistent pain in general has a high socioeconomic burden, can be very debilitating, with reduced physical activity, and social isolation [11].

Quantitative Sensory Testing (QST) is a reliable methodology to objectively and accurately assess changes in sensory functioning beyond that of self-reported pain assessments. This technique enables measurement of sensory sensitivities, including that for pain and different sensory modalities [12]. A validated QST protocol was originally developed for testing the somatosensory system in adults [12–15], and the QST protocol was validated in healthy children from 5 years and older [13]. A few studies using QST in children with JIA showed an increased sensitivity to painful mechanical and thermal stimuli at the thenar eminence and knee; even in absence of markers of disease activity, and for a prolonged period of time [16, 17]. However, each joint is differently innervated and sensitivity levels differ depending on the tested location (e.g., the face yields different values from the hand) [12, 13]. In JIA, the knee is the most frequently affected joint. It is currently unknown whether and how different sensory thresholds at the knee are affected by JIA.

In this study, we tested an extensive set of sensory modalities in the knees of Dutch Patients with JIA to test the feasibility of performing the QST across ages 6–18, and provide novel reference data of sensory thresholds for the knee of children aged between 9 and 18. To this end, a validated QST battery was used in children with JIA and healthy controls.

## Materials & methods

### Study design

Patients who visited the outpatient clinic of the pediatric rheumatology department were screened for eligibility. Patients were included if the following criteria were met: age between 6 and 18 years, able to speak and understand the Dutch language, JIA according to the International League Against Rheumatism (ILAR) criteria, inflammation in the knee, and a general pain score of 10 or more on a 0-100 mm patient Visual Analogue Scale (VAS). Exclusion criteria were: damaged skin on one or both knees, severe psychiatric co-morbidity, or active substance abuse. The study was designed as an observational cross-sectional case-control study. Cases and healthy controls were matched by age (within 1 year) and gender. The study was conducted in accordance with the Declaration of Helsinki (7th amendment, October 2013), and approved by the ethics review board (protocol number 13–577). Written informed consent was obtained from all participants and from their parents or custodial caregiver.

### Clinical parameters

Patient characteristics were derived from the medical records. As part of standard of clinical care, a complete physical examination was performed by the rheumatologist, comprising the assessment of the number of active joints, Physician Global Assessment, and erythrocyte sediment rate (ESR).

In addition, the Childhood Health Assessment Questionnaire (CHAQ) was provided by patient and/or parent, from which the disease severity score on a 0–100 scale was derived (with 0 being no disease activity and 100 being the most severe activity) [18]. From these measurements, the Juvenile Arthritis Disease Activity Score (JADAS) was calculated as described previously [19]. VAS scales are validated for children aged 5 years or older [20].

### Quantitative sensory testing

QST was performed by trained personnel according to the QST protocol for children as described previously [13]. In all patients, the tests were carried out on the affected knee and the unaffected control knee. In healthy controls the right knee was tested. Before each actual test, a practice trial was performed, to ascertain that the subject understood the test. Typically, a test took just over 40 minutes to complete. When an affected as well as an unaffected knee were tested, the total time could go beyond 90 minutes. The QST protocol consisted of the following tests:

1. Thermal detection thresholds for cold (cold detection threshold; CDT) and warm (warm detection threshold; WDT) stimuli. Thermal stimuli were applied using a thermal stimulation device with a 3 × 3 cm2 surface Peltier-type thermode (Pathway, Medoc®, Israel). Thermal detection thresholds were determined by the mean of three consecutive stimuli.
2. Thermal pain thresholds for cold (cold pain threshold; CPT) and hot stimuli (heat pain threshold; HPT). Stimuli were either continuously increasing or decreasing in temperature until the patient presses a button to indicating the detection of a change from a neutral sensation to a painful hot/cold sensation. Thermal pain thresholds were determined by the mean of three consecutive stimuli.
3. Thermal sensory limen (TSL) was determined using a ramped increase from 32 degrees Centigrade upwards. The subject was asked to press a button as soon as the subject experiences a change of temperature to a warm sensation. Thereafter the temperature would decrease, and the subject was asked to press as soon as a cold sensation was perceived. This scheme was repeated for a total of six times and the TSL was determined based on the difference between the means of the two extremes in temperature.
4. Mechanical detection threshold (MDT) is determined using a standardized set of modified von Frey hairs (Optihair2-Set, Marstock Nervtest, Germany) that exert forces between 0.25 and 512 mN. The MDT was determined as the geometric mean of five consecutive series of ascending and descending stimulus intensities.
5. Mechanical pain threshold (MPT) is determined using pinprick stimuli using a set of seven custom-made (DFNS, Germany) weighted pinprick stimulators (flat contact area of 0.2 mm diameter) that exert forces between 8 and 512 mN. The MPT is the geometric mean of five consecutive series of ascending and descending stimulus intensities.
6. Wind-up ratio (WUR) compares the numerical ratings of five series of a) a single pinprick stimulus (mostly 256 mN, depending on the pinprick threshold) with five series of b) 10 repetitive pinprick stimuli at a 1/s rate. Each series of stimuli is applied within an alternating surface area of 1 cm2, immediately followed by an average numerical pain rating of the preceding series. The WUR is calculated as the averaged ratio: b/a.
7. Vibration detection threshold (VDT) is determined using a Rydel–Seiffer 64 Hz tuning fork placed over the head of the fibula. Vibration threshold is determined with three series of diminishing stimulus intensities. The average of the three detection thresholds is used as the VDT.
8. Pressure pain threshold (PPT) is determined with a pressure gauge device (FDN100, Wagner Instruments, USA) with a probe area of 1 cm^2^ that exerts pressure up to 10 kg/cm^2^. Three series of ascending stimulus intensities are applied as a slowly increasing ramp of 0,5 kg/cm^2^ per second and the mean is taken as the PPT.
9. Mechanical pain / dynamic mechanical allodynia (ALL) assesses pain in response to stroking light touch (CW = cotton wisp; QT = cotton wool tip; BR = brush). The three tactile stimuli are applied five times each with a single stroke of approximately 1–2 cm in length over the skin. This is pseudo-randomly alternated with pinprick stimuli of various forces as described above.

All tests were executed in accordance with the protocol (see complementary material).

### Sample size calculation and feasibility criteria

Sample size was calculated based on published data on QST measurements in Patients with JIA [9, 16]. Data were assumed to be normally distributed. From the results of Cornelissen [16], it can be found that having 60 patients, and 151 healthy controls, the median difference in absolute QST values is 4.0 for the CPT. Converting the IQR to a pooled SD, with the SD being estimated to be 0.75*IQR yields a pooled SD of 4.40. Dividing the median difference score by the pooled SD results in an effect size (Cohen’s d) of 0.9. Using a 2-tailed T-test with two groups with independent means, a total sample size of 42 is obtained, divided in a patient group and control group of both 21.

In order to detect a 38.5% difference in cold pain thresholds between the affected body regions in patients with JIA and control body regions in healthy controls with a standard deviation of 42.3%, a total sample size of 21 per age group was needed. Two age groups were defined: age 6 to 9, and 9 to 18. Sample size calculations were performed using G*Power 3 for a 2-tailed independent T-test. We assumed a type-I-error of 0.05 and a type-II-error of maximal 0.20. Based on the initial results from Blankenberg et al. [9] QST testing was deemed feasible if less or equal than 10% of subjects per age group failed to complete the procedure.

### Statistics

Data are represented as median and IQR, or mean and SD where appropriate. For the QST tests, one-way ANOVA with Tukey’s post hoc analysis was performed for parametric data, and Kruskall-Wallis test for non-parametric data. For the statistical analysis, R version.3.3.2 was used. Data were log-transformed where appropriate and z-values were calculated [21].

## Results

In the age group of 6 to 9 years, participants were not able to complete the tests because of lack of understanding or inadequate attention span. After five inclusions within this age range, three subjects had not been able to complete the QST protocol. In this age group, the endpoint of feasibility had been reached (3 incomplete tests out of 21 yields 14% failure). Therefore we decided to only include and analyze children at an age of 9–18 years from that point on.

The demographic and clinical data of patients with JIA of the older age group (9–18 years, *N* = 16) are shown in Table 1. The duration of the disease ranged from 3 to 196 months. The mean age was 15+/− 2 years, and the mean time between diagnosis and study inclusion was 74 months. The mean JADAS score of the patients was 10+/− 5.6 and the (CHAQ) pain score averaged 54+/− 30 (on a scale of 0–100). All patients had pain duration of more than 2 months.

**Table 1:** Patient characteristics

Number of subjects aged 9–18	16
Mean age (years) (SD)	14.8 (2.1)
Female gender (%)	75
Type JIA	
- persistent oligoarthritis (%)	*57.1*
- extended oligoarthritis (%)	*7.1*
- RF negative polyarthritis (%)	*28.6*
- RF positive polyarthritis (%)	*7.1*
Duration since diagnosis (months) (SD)	74.0 (67.3)
JADAS score (SD)	10.2 (5.6)
CHAQ pain (SD)	54.3 (29.5)
Number of affected joints (SD)	2.7 (2.7)
ESR (SD)	24.4 (18.9)
medication used (%)	
- NSAID	64.3
- Methotrexate	35.7
- Humira	14.3
- Triamcinolon	7.1
- Leflunomide	7.1

Overview of clinical data of patients included (*N* = 16); *SD* Standard deviation, *JIA* juvenile idiopathic arthritis, *RF* Reumatoid Factor, *JADAS* Juvenile Arthritis Disease Activity Score, *CHAQ* Childhood Health Assessment Questionnaires, *ESR* Erythrocyte sedimentation rate

### Pain threshold

**Fig. 1:**
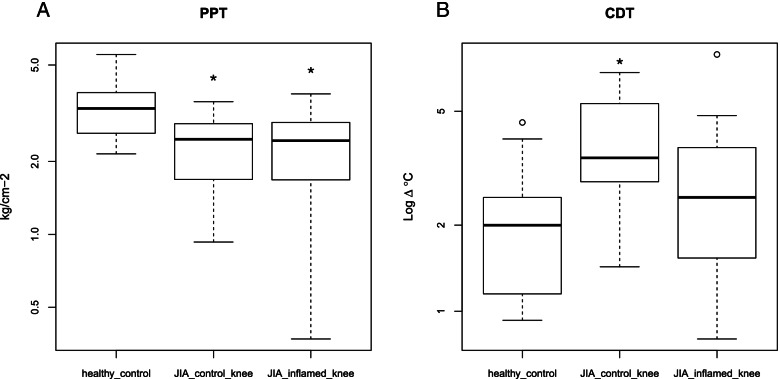
Sensory deficits were measured using the QST. (**A**) PPT were significantly lower at the control and affected knee of JIA patient in comparison with healthy controls (**B**) Sensory detection thresholds were significantly higher at the control knee of JIA patient compared to healthy control knee, as well as affected knee. PPT: pressure pain threshold, CDT: cold detection threshold. Higher value means less sensitive to cold detection. * *p* < 0.05

Pressure pain thresholds of the affected knee of Patients with JIA were significantly lower than that of the knee of healthy control subjects (df = 2, F = 5.176 Pr(>F) = 0.00979, *p* = 0.015). Moreover, even the unaffected unaffected control knee PPT thresholds of patients with JIA were lower than at the knees from healthy control subjects (*p* = 0.033). Pressure pain thresholds did not differ significantly between the affected and the unaffected knee of the patients with JIA (*p* = 0.93). (see Fig. 1A).

Heat pain thresholds (*p* = 0.57), cold pain thresholds (*p* = 0.46), or mechanical pain thresholds (*p* = 0.67) of the affectedaffected knee of patients with JIA did not differ significantly from unaffected unaffected knees or knees of healthy control subjects (see Fig. 1 of supplementary material).

### Sensory detection thresholds

Cold detection thresholds were significantly higher in the unaffected knee of patients with JIA compared to knees of healthy control subjects (*p* < 0.01; Fig. 1B). In contrast, the affected knee of JIA patient did not differ from healthy control knees. Warm detection thresholds (*p* = 0.73), temperature sensory limen (*p* = 0.1), mechanical detection thresholds (*p* = 0.83), allodynia (*p* = 0.46), vibration detection thresholds (*p* = 0.3) and windup ratio (*p* = 0.31) did not significantly differ between the groups (see Fig. 1 of supplementary material).

### Associations between perceived pain and QST measures

**Fig. 2:**
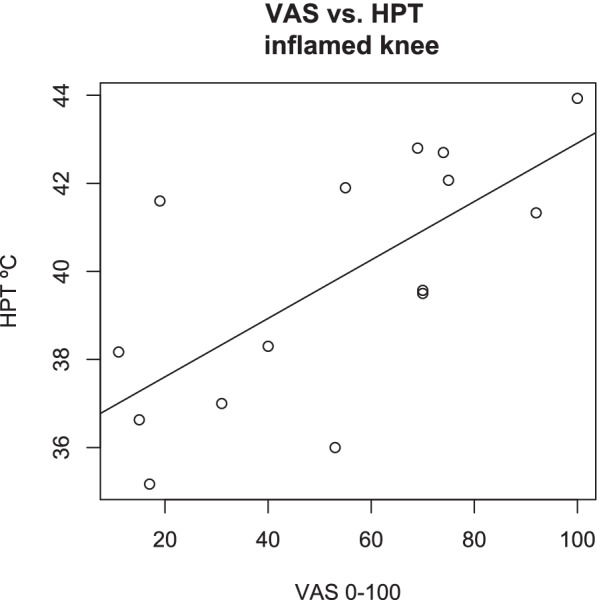
Correlation between HTP and VAS pain. VAS: visual analog scale; HPT: heat pain threshold, *n* = 14. *p < 0.001

Next we tested for correlations between reported pain and QST measures. Heat pain threshold (HPT) and patient VAS were correlated (Pearson’s r = 0.70, *p <* 0.01; Fig. 2). All other QST modalities did not correlate with the magnitude of reported pain (Table 2).

**Table 2:** Correlation between QST tests and reported clinical pain

	Reported pain
**CDT**	0.25 (0.38)
**WDT**	0.32 (0.25)
**TSL**	0.16 (0.57)
**CPT**	0.31 (0.26)
**HPT**	0.70 (< 0.01^a^)
**MDT**	0.12 (0.67)
**MPT**	0.06 (0.84)
**VDT**	0.08 (0.78)
**PPT**	0.34 (0.24)
**WUR**	0.10 (0.73)
**ALL**	0.38 (0.20)

From all tested QST measures HPT correlated with reported clinical pain in Patients with JIA. Depicted values are Pearson’s R (*p-*level). *CDT* Cold detection threshold, *WDT* Warm detection threshold, *TSL* Thermal sensory limen, *CPT* Cold pain threshold, *HPT* Heat pain threshold, *MDT* mechanical detection threshold, *MPT* Mechanical pain threshold, *VDT* Vibration detection threshold, *PPT* Pressure pain threshold, *WUR* Wind-up ratio, *ALL* Allodynia

^a^ Significant at 0.01

## Discussion

JIA is a well-known childhood disease with chronic pain being one of the symptoms. Objective quantification of changes in somatosensory processing such as QST in children with JIA aids in better understanding pain in a research setting for this disease. In response to the primary outcome, we show here that the German Research Network on Neuropathic Pain validated QST protocol [12] is feasible in Dutch children aged 9 years and up, but not in children between 6 and 8 years of age.

Importantly, we identified that the pressure pain threshold is reduced at the affected and unaffected knee of patients with JIA compared to healthy controls, whilst other pain thresholds did not differ from healthy control. Unexpectedly, the cold detection threshold was reduced only in the unaffected knee of patients with JIA, while no significant differences were detected in the affected knee of patients with JIA compared to controls. Possibly, the study population was too small to show significant CDT change in the affected knee. In conclusion, in our JIA patient population, Cold Detection Test and Pressure Pain Test were significantly different between patients with JIA and healthy controls.

We detected a reduction in PPT at both the affected and unaffected unaffected knee of Patients with JIA, indicating a generalized enhanced pain sensitivity (of hyperalgesic response) to pressure stimuli. Although others also have found that a unilateral inflammation induces a bilateral reduction in PPT in patients with JIA [10], we now confirm these findings with validated and standardized QST measures. The question rises why in both the affected and unaffected knee changes in PPT were observed. In adult rheumatoid arthritis patients, pain symptoms often arise before clinical manifestation of the arthritis [22], thus by analogy the possibility exists that some of patients with JIA had subclinical inflammation at the unaffected knee causing the reduction in PPT. Alternatively, in patients with JIA systemic inflammatory responses may cause bilateral reduction in PPT. For example, experimental systemic inflammation induced with an i.v. bolus with lipopolysacharide induced a generalized reduction in pressure pain threshold in healthy volunteers [23]. Similarly, in patients with a unilateral neuropathy, pain sensory abnormalities, in particular for pressure pain threshold, were also observed at the non-affected site [24]. It has been proposed that spreading of spinal glial activation and central sensitization induces bilateral alterations in sensory detection thresholds [25] in preclinical mouse studies and pilot data in patients with JIA [26]. However, it remains to be determined whether such spinal changes occur in patients with JIA.

Importantly, the most frequent site of involvement in JIA is the knee, and changes in sensory modalities specifically in the knee joint have not been studied in the context of JIA.

An earlier study [16] has included QST measures of the knee of patients with JIA, however, these knee QST datasets were combined with datasets of the ankle joint to compare active (*n* = 17) and in-active joints (*n* = 12). Whether the combined knee and ankle joint thresholds differed from that of healthy controls was not reported. In this study, we assessed sensory changes specifically at the knee of healthy controls and patients with JIA with an affected and unaffected knee. We observed that only two sensory modalities (CDT and PPT) at the knee were affected compared to healthy control knee. No significant differences were observed between the affected and unaffected knee, similar to what was previously observed when knee and ankle joints were combined [16]. An earlier study by Cornelissen et al. [16] showed significant differences for all QST modalities when the inflammation of the ankle, knee or face was compared to the thenar eminence of a healthy control. However, QST data obtained from different joints is difficult to compare, as sensory thresholds of each joint are different. For example, the density of nerve endings at the thenar eminence is higher than at the knee, reducing the discriminatory sensitivity at the knee compared to the hand [27].

It is worth mentioning that apart from HPT, in this cross-sectional study, no QST modalities had a significant correlation with the magnitude of the reported pain. This is in accordance with the findings of Arnstad et al. [17] It is well known that the perception of pain is the result of complex biopsychosocial processing. Whether or not such a correlation between QST and reported magnitude of pain exists should be investigated in future longitudinal studies, or larger cross-sectional studies.

One limitation of this study is that the average duration of JIA was 6 years at the time of study. Thus the data represent sensory changes associated with chronic inflammation. Future studies could focus on sensory changes at the onset of rheumatic disease and how these changes correlate to pain levels during later stages of the disease. Also, in this population, medication was used extensively and ranged from NSAIDs to DMARDs. These drugs are known to have a dampening effect on pain [28], and as such could have affected pain VAS score and possible even QST measures.

In conclusion, this study provides information that in Dutch children, the complete QST protocol is only feasible in the age group from 9 years and older, because children younger than 9 years were not able to complete the more extended DFNS QST test battery. In future studies, a reduced set of QST tests containing at least pressure pain thresholds and cold detection thresholds could prove to be better suited to the pediatric arthritis population, with the purpose of identifying patients with persistent changes in somatosensory processing in longitudinal studies. This would also reduce the 40 minutes test to roughly 10 minutes instead.

## Supplementary Information

**Additional file 1.** CDT, cold detection threshold; WDT, warm detection threshold; TSL, thermal sensory limen; CPT, cold pain threshold; HPT, heat pain threshold; MDT, mechanical detection threshold; MPT, mechanical pain threshold; VDT, vibration detection threshold; PPT, pressure pain threshold; WUR, wind-up ratio; ALL, allodynia. * Significant at 0.05.
