# Supplementary material for: Feasibility of quantitative sensory testing in juvenile idiopathic arthritis
Source: Pediatr Rheumatol Online J. 2022 Aug 9;20:63. doi: 10.1186/s12969-022-00715-5 (PMC9364560; doi:10.1186/s12969-022-00715-5)
Supplement: Supplementary file 1 — Additional file 1. CDT, cold detection threshold; WDT, warm detection threshold; TSL, thermal sensory limen; CPT, cold pain threshold; HPT, heat pain threshold; MDT, mechanical detection threshold; MPT, mechanical pain threshold; VDT, vibration detection threshold; PPT, pressure pain threshold; WUR, wind-up ratio; ALL, allodynia. * Significant at 0.05. [file 12969_2022_715_MOESM1_ESM.docx]

**SUPPLEMENTARY MATERIAL**

**Figure 1:**


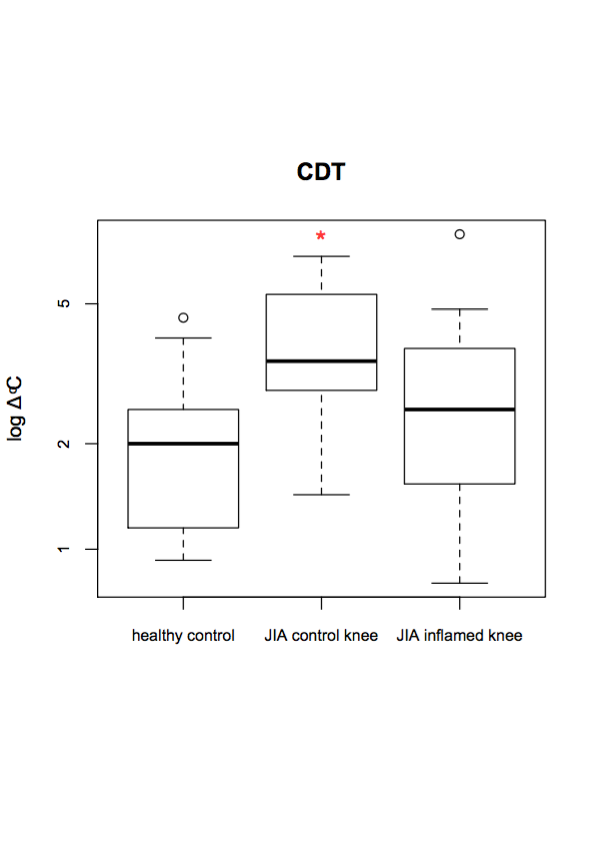
**
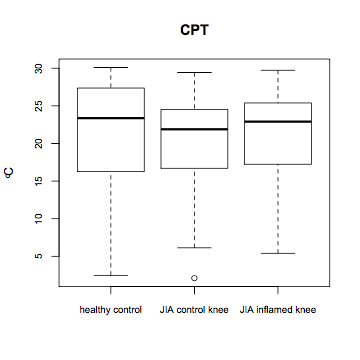

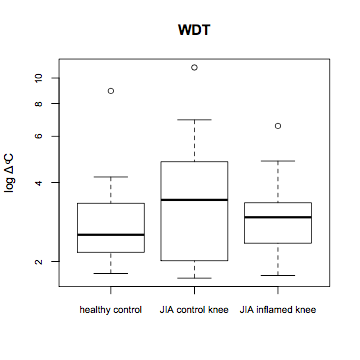

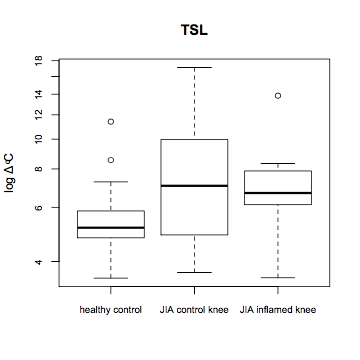

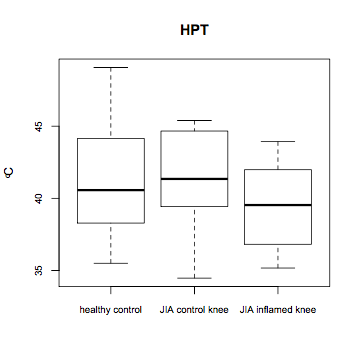

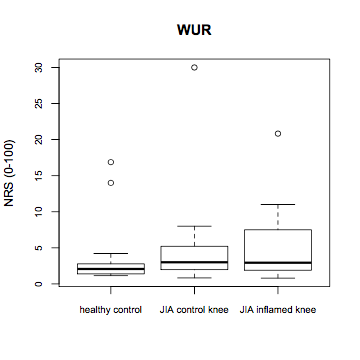

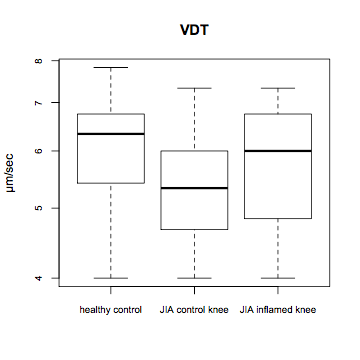

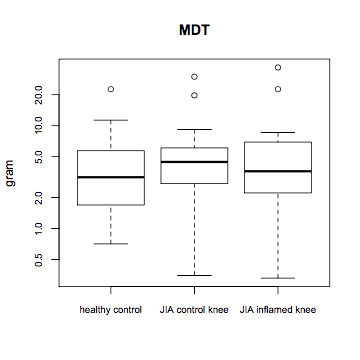
**

**Legend:** CDT, cold detection threshold; WDT, warm detection threshold; TSL, thermal sensory limen; CPT, cold pain threshold; HPT, heat pain threshold; MDT, mechanical detection threshold; MPT, mechanical pain threshold; VDT, vibration detection threshold; PPT, pressure pain threshold; WUR, wind-up ratio; ALL, allodynia. * Significant at 0.05

**
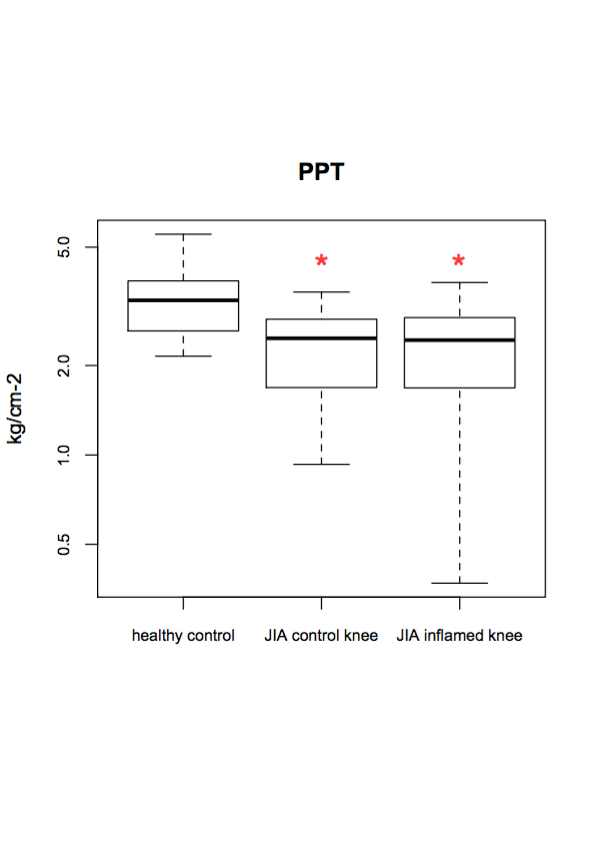
**
